# The new method of ZnIn_2_S_4_ synthesis on the titania nanotubes substrate with enhanced stability and photoelectrochemical performance

**DOI:** 10.1038/s41598-023-48309-9

**Published:** 2023-12-02

**Authors:** D. Roda, K. Trzciński, M. Łapiński, M. Gazda, M. Sawczak, A. P. Nowak, M. Szkoda

**Affiliations:** 1 Department of Chemistry and Technology of Functional Materials, Faculty of Chemistry, Gdańsk University of Technology, Narutowicza 11/12, 80-233 Gdańsk, Poland; 2 Advanced Materials Center, Gdańsk University of Technology, Narutowicza 11/12, 80-233 Gdańsk, Poland; 3 Faculty of Applied Physics and Mathematics, Gdańsk University of Technology, Narutowicza 11/12, 80-233 Gdańsk, Poland; 4 Centre for Plasma and Laser Engineering, The Szewalski Institute of Fluid Flow Machinery, Fiszera 14, 80-231 Gdańsk, Poland

**Keywords:** Energy science and technology, Electrochemistry, Energy, Green chemistry

## Abstract

In this work, ZnIn_2_S_4_ layers were obtained on fluorine doped tin oxide (FTO) glass and TiO_2_ nanotubes (TiO_2_NT) using a hydrothermal process as photoanodes for photoelectrochemical (PEC) water splitting. Then, samples were annealed and the effect of the annealing temperature was investigated. Optimization of the deposition process and annealing of ZnIn_2_S_4_ layers made it possible to obtain an FTO-based material generating a photocurrent of 1.2 mA cm^−2^ at 1.62 V vs. RHE in a neutral medium. In contrast, the highest photocurrent in the neutral electrolyte obtained for the TiO_2_NT-based photoanode reached 0.5 mA cm^−2^ at 1.62 V vs. RHE. In addition, the use of a strongly acidic electrolyte allowed the generated photocurrent by the TiO_2_NT-based photoanode to increase to 3.02 mA cm^−2^ at 0.31 V vs. RHE. Despite a weaker photoresponse in neutral electrolyte than the optimized FTO-based photoanode, the use of TiO_2_NT as a substrate allowed for a significant increase in the photoanode's operating time. After 2 h of illumination, the photocurrent response of the TiO_2_NT-based photoanode was 0.21 mA cm^−2^, which was 42% of the initial value. In contrast, the FTO-based photoanode after the same time generated a photocurrent of 0.02 mA cm^−2^ which was only 1% of the initial value. The results indicated that the use of TiO_2_ nanotubes as a substrate for ZnIn_2_S_4_ deposition increases the photoanode's long-term stability in photoelectrochemical water splitting. The proposed charge transfer mechanism suggested that the heterojunction between ZnIn_2_S_4_ and TiO_2_ played an important role in improving the stability of the material by supporting charge separation.

## Introduction

The continuous increase in energy consumption and finite reserves of fossil fuels are the reason for the search for alternative energy sources. This is one of the biggest challenges facing society today. Converting solar energy into chemical fuels is one of the most promising solutions for fulfilling this demand^1,2^. Production of hydrogen fuel using photoelectrochemical cells (PEC) seems to be a very good and ecological way to develop renewable energy sources. Generally, the PEC process can be divided into three steps to convert solar energy into H_2_ generation. In the first step, charge carriers are generated when photoelectrodes absorb photons whose energy is equal to or higher than the band gap energy. In the second step, the electron–hole pair are separated. Finally, electrons or holes (depending on the type of electrode) participate in redox reactions at the electrode surface^3^. The search for suitable materials active in visible light is based on the following factors: (1) photoactive materials should be characterized by a relatively narrow energy gap (1.9–3 eV), (2) appropriate band structure (conduction band/valence band meeting the oxidation and reduction potential of water), (3) fast charge separation, (4) undisturbed charge transport and (5) photostability^4^.

Since the work of Fujishima and Honda on photoelectrochemical water decomposition using TiO_2_ monocrystals^5^, many other semiconductor materials have been investigated for the same application. Along with TiO_2_, which is still widely used, many other metal oxides have been explored as photoanode materials for photoelectrochemical water splitting (i.e. WO_3_^6,7^, Fe_2_O_3_^8,9^, CuO^10,11^, ZnO^12^). The biggest problem regarding the application of such compounds is that oxide semiconductors are characterized by wide energy gaps, which makes them active under UV light (λ < 389 nm), which is only 5% of the solar spectrum reaching the Earth^13,14^. As opposed to metal oxides, many metal sulfides have narrow energy gaps suitable for the absorption of visible light^15,16^. Metal sulfides, in addition to the advantages mentioned above, unfortunately, have several disadvantages that limit their use, such as low electron and hole separation efficiency and slow water oxidation kinetics^18^, lack of suitable oxidation and reduction potentials to achieve complete water decomposition or vulnerability to oxidation in air or aqueous solutions^19,20^. One of the most commonly studied for PEC water splitting among metal sulfides is CdS, active under visible light (band gap 2.4 eV)^21^. However, CdS-based photoelectrodes easily undergo photocorrosion due to the unstable valence band composed of S 3p orbitals^22,23^. The problem of metal sulfide instability has been tried to be solved, for example, by morphology and grain control^24^, but the obtained materials had low photocatalytic activity. It has been proven that multicomponent metal sulfides exhibit photocatalytic activity^25,26^. One of the most studied materials in this group is CdIn_2_S_4_^17^. Despite the efforts made so far to enable the use of this compound in photoanodes, there are many problems have not yet been overcome, such as high surface recombination^27^. Besides CdS, a well-known sulfide semiconductor is ZnS, which is active under UV light due to its wide energy gap^28^. Accordingly, another multicomponent sulfide, ZnIn_2_S_4_, is the next candidate as a material active under visible light for use in photoanodes. Zinc indium sulfide (ZnIn_2_S_4_ ZIS), which belongs to the group of ternary compounds with the general formula AB_2_X_4_, is one of the most promising semiconductor photocatalysts among metal sulfides^29^. It is characterized by 2D sheet structures and stands out for its many advantages in various fields such as charge storage, photocatalytic water splitting, and CO_2_ photoreduction^30–33^. Until now, ZnIn_2_S_4_ has been obtained mainly in the form of powder^34–36^ or films on the FTO glass^37–39^. FTO is one of the most commonly used substrates for photoelectrodes^6,8,18^. It has good thermal stability and its resistance does not increase during annealing even at 450 °C^40^. Moreover, FTO sheets with low sheet resistance (7–8 Ω sq^−1^) are widely available commercially. Many articles on the use of ZnIn_2_S_4_ in photoelectrochemical water splitting do not report at all results on the stability of the electrodes^38,41,42^. This suggests that the ZnIn_2_S_4_-based materials described so far do not exhibit good photoactivity during long-term testing. The use of a material that is photochemically active could reduce the impact of negative factors on the photoanode's stability through the formation of a heterojunction, which has already been described in the literature^43,44^. TiO_2_ nanotubes are therefore an excellent potential substrate, as they can simultaneously act as a substrate with a greatly developed surface area and form a heterojunction with ZnIn_2_S_4_.

Titanium dioxide (TiO_2_) is among the most studied photocatalysts. It has unique properties such as chemical stability, non-toxicity, and bio-compatibility^45^. One of the most studied TiO_2_ nanostructures are nanotubes because their chemical and physical properties can be controlled to the greatest extent^46^. TiO_2_ nanotubes (TiO_2_NT) obtained by anodization are formed directly on titanium, which acts as the substrate and current collector. Therefore, there is no need to add a binder, which usually increases the resistance. TiO_2_ nanotubes obtained by anodization on titanium represent a promising substrate for the deposition of other photoactive materials^47–49^.

While both substrates are commonly used in photoelectrochemical research^6,8,47,48^, a direct comparison of ZnIn_2_S_4_ performance on these substrates is lacking, making it essential to fill this gap for a comprehensive understanding of the material's behavior in practical applications. Thus, comparative photoelectrochemical (PEC) studies of ZnIn_2_S_4_ on anodized TiO_2_ nanotubes and fluorine-doped tin oxide (FTO) substrates may be of significant importance in the field of photoelectrochemical research. Understanding how the same material behaves on different substrates allows for a comprehensive evaluation of its properties and potential in renewable energy applications. The choice of substrate can profoundly affect the stability and long-term performance of photoelectrochemical devices. By conducting comparative studies, one can identify the substrate that provides better stability for ZnIn_2_S_4_-based photoanodes, enabling the development of more durable and practical devices.

This study presents a comparison of the photoelectrochemical properties of ZnIn_2_S_4_ layers deposited on FTO and TiO_2_ nanotubes (TiO_2_NT). ZnIn_2_S_4_ was obtained using a hydrothermal process previously described in the literature^38^. The materials obtained by processes lasting for 6 and 12 h were also compared. Moreover, the ZnIn_2_S_4_ layers on FTO and TiO_2_NT were annealed at different temperatures and tested as photoanodes for water oxidation. Photoelectrochemical studies were performed in a three-electrode system under simulated solar light illumination. The differences in the active layer properties obtained on TiO_2_ nanotubes and FTO were studied using a series of structural, morphological, and optical characterization. Photoelectrochemical measurements made it possible to determine the photoelectrochemical activity of the materials obtained and to compare their stability during illumination.

The procedures of substrate preparation and Ti anodization were described in supporting information (SI). The materials characterization section was also described in SI. The ZnIn_2_S_4_ layers were prepared directly on FTO and TiO_2_NT substrates using a hydrothermal process. Details are presented in SI. The photoanodes obtained in the first, hydrothermal stage were then annealed in air atmosphere at temperatures of 300, 400, and 500 °C. The applied designations of all the types of photoanodes, along with the parameters of the processes for obtaining them, are shown in Table 1.

**Table 1:** Inventory of the obtained electrodes with the parameters of the obtaining processes.

Material	Substrate	Time of hydrothermal process [h]	Annealing temperature [°C]
FTO/ZIS_6_	FTO	6	–
FTO/ZIS_6_-O_300_	FTO	6	300
FTO/ZIS_6_-O_400_	FTO	6	400
FTO/ZIS_6_-O_500_	FTO	6	500
FTO/ZIS_12_	FTO	12	–
FTO/ZIS_12_-O_300_	FTO	12	300
FTO/ZIS_12_-O_400_	FTO	12	400
FTO/ZIS_12_-O_500_	FTO	12	500
TiO_2_NT/ZIS_6_	TiO_2_NT	6	–
TiO_2_NT/ZIS_6_-O_300_	TiO_2_NT	6	300
TiO_2_NT/ZIS_6_-O_400_	TiO_2_NT	6	400
TiO_2_NT/ZIS_6_-O_500_	TiO_2_NT	6	500
TiO_2_NT/ZIS_12_	TiO_2_NT	12	–
TiO_2_NT/ZIS_12_-O_300_	TiO_2_NT	12	300
TiO_2_NT/ZIS_12_-O_400_	TiO_2_NT	12	400
TiO_2_NT/ZIS_12_-O_500_	TiO_2_NT	12	500

## Results and discussion

**Figure 1:**
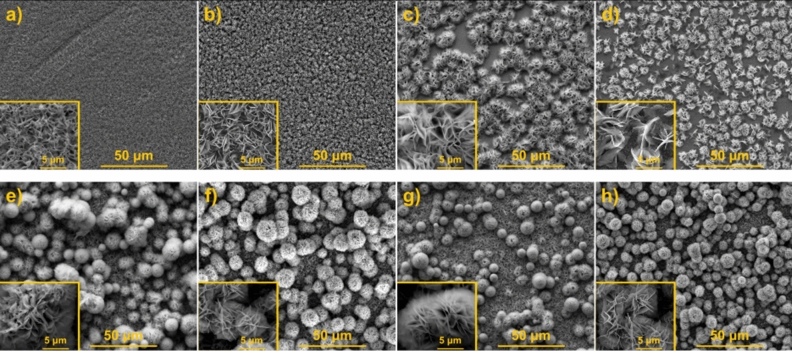
SEM images of (**a**) FTO/ZIS_6_, (**b**) FTO/ZIS_6_-O_300_, (**c**) FTO/ZIS_6_-O_400_, (**d**) FTO/ZIS_6_-O_500_, (**e**) TiO_2_NT/ZIS_6_, (**f**) TiO_2_NT/ZIS_6_-O_300_, (**g**) TiO_2_NT/ZIS_6_-O_400_ and (**h**) TiO_2_NT/ZIS_6_-O_500_.

The morphologies of the prepared samples were examined by SEM. Figure 1 shows the micrographs of ZnIn_2_S_4_ layers obtained by the 6 h process on FTO and TiO_2_NT (Fig. 1a–d,e–h, respectively) before and after annealing. The resulting layers significantly differ depending on the substrate on which they were deposited. In the case of FTO, ZnIn_2_S_4_ was obtained uniformly over the entire surface. Annealing of the layers on FTO at 400 and 500 °C (Fig. 1c,d, respectively) results in the formation of spherical aggregates of deposited material between which the substrate surface was exposed. In the case of the layers on TiO_2_NT, marigold-like ZnIn_2_S_4_ microspheres described earlier in literature^50^ can be observed even before the annealing (Fig. 1e). After annealing on TiO_2_NT, although the deeper layers of the material were exposed, the substrate surface was not visible (see Fig. 1f–h). It is seen that the effect of temperature on surface morphology was not as significant for the layers on TiO_2_NT as for these on FTO. The use of TiO_2_ nanotubes enabled better coverage of the substrate surface with ZIS material.

**Figure 2:**
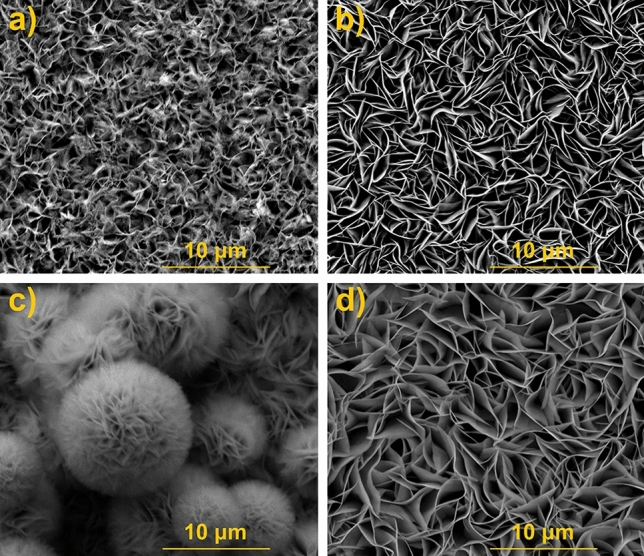
SEM images of (**a**) FTO/ZIS_6_, (**b**) FTO/ZIS_12_, (**c**) TiO_2_NT/ZIS_6_ and (**d**) TiO_2_NT/ZIS_12_.

SEM images of the layers obtained by the 12 h hydrothermal process before and after annealing are shown in Figure S1. Neither the type of substrate nor the annealing temperature significantly affects the morphology of the resulting layers. No areas of the exposed substrate surface are visible on both FTO and TiO_2_NT except FTO/ZIS_12_-O_500_. After annealing the layer on FTO at 500 °C, the formation of aggregates of deposited material can be observed (Fig. S1d). Figure 2 presents a comparison of the SEM micrographs of the layers after 6 and 12 h hydrothermal processes on both substrates. As can be seen in Fig. 2b, the 12 h hydrothermal process leads to vertically aligned flakes on FTO that are more ordered than the layers obtained by the 6 h process on the same substrate (Fig. 2a). Regarding morphology, TiO_2_NT/ZIS_6_ and TiO_2_NT/ZIS_12_ differ significantly from each other (Fig. 2c,d). The marigold like spheres structure is observed for TiO_2_NT/ZIS_6_, while TiO_2_NT/ZIS_12_ materials look rather like nanoflakes.

**Figure 3:**
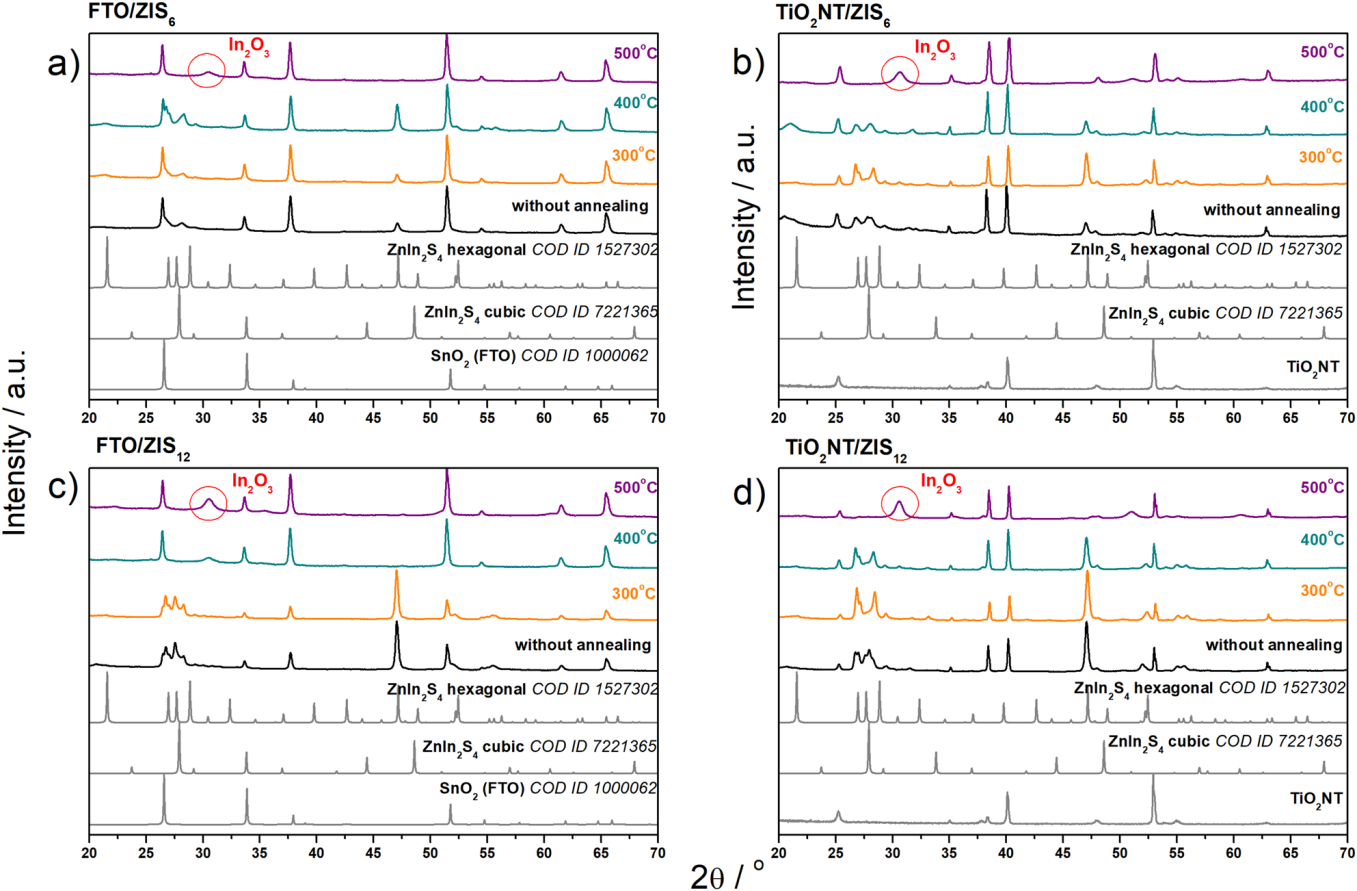
XRD patterns of (**a**) FTO/ZIS_6_, (**b**) TiO_2_NT/ZIS_6_, (**c**) FTO/ZIS_12_ and (**d**) TiO_2_NT/ZIS_12_ photoanodes before and after annealing at 300, 400 and 500 °C.

The crystal structure of the prepared ZnIn_2_S_4_ layers before and after annealing at different temperatures has been studied by X-ray diffraction (XRD) technique, as shown in Fig. 3. In the patterns for materials before annealing, regardless of the length of the synthesis, only the XRD reflections of hexagonal and cubic ZnIn_2_S_4_ structures, as well as these of the substrates can be seen. Therefore, it is confirmed that the ZnIn_2_S_4_ material was successfully obtained on completely different substrates using the same procedure. In all cases, the reflections corresponding to the hexagonal structure of ZnIn_2_S_4_ are relatively higher than those of the cubic one. The only difference between the material synthesized at 6 h and 12 h is the intensity ratio for the peaks at ~ 46° and ~ 53°, which correspond to the plane (2 $\overline{1 }$ 0) of hexagonal ZnIn_2_S_4_ and the substrate (both FTO and TiO_2_NT), respectively. For the shorter time, the substrate surface is more exposed for both substrates, so the peak intensity at ~ 53° is higher than for the longer synthesis time. Consequently, the intensity of the peak corresponding to ZnIn_2_S_4_ increases with better surface coverage in the 12 h process. The reflections seen in the patterns of the layers processed for 12 h in comparison to those for 6 h are higher and narrower which means that the layer is thicker and composed of larger crystallites. As the annealing temperature increases, the reflections at 2θ equal to 27° and 46.5° corresponding to the (101) and (1 $\overline{1 }$ 0) planes of the hexagonal structure are no longer visible. The reflection at 28° from the (311) plane of the cubic structure also disappears. This indicates the decomposition of crystalline ZnIn_2_S_4_ during the annealing of the material at higher temperatures. It is known that annealing of indium sulfides at temperatures above 400 °C leads to the formation of In_2_O_3_^51^. Thus, annealing at 500 °C of all materials, results in the formation of In_2_O_3_, from which a clear, characteristic reflection is seen at 30.6° (JCPDS No. 71-2195)^37^. As the annealing temperature increases, the relative intensity of the peaks corresponding to SnO_2_ in XRD patterns for FTO-based materials also increases (see Fig. 3a,c). This confirms that annealing results in the exposure of the substrate surface, as the signal from the substrate is more intense.

The average crystal size of ZnIn_2_S_4_ layers on FTO and TiO_2_NT was calculated from XRD measurements using the Scherrer equation, and the results were presented in Table S1. To compare the effect of temperature on crystallite size on the two substrates, the peak at 62.5° was chosen for the calculations because it is visible in the diffractograms for all materials. ZnIn_2_S_4_ grains in layers on FTO were characterized by sizes of 41–44 nm. In contrast, layers with much higher grain sizes were obtained on TiO_2_NT: 65–133 nm. This variation in crystal size could stem from the distinct properties of the substrates, which can influence the nucleation and growth of the ZnIn_2_S_4_ crystalline structure. In the case of FTO-based photoanodes, the length of the hydrothermal process and the annealing temperature had no significant effect on the change in crystallite size. Whereas, ZnIn_2_S_4_ grains in layers deposited on TiO_2_NT obtained in a 12 h hydrothermal process were characterized by sizes larger by about 31 nm than in the case of shorter deposition (6 h). Increasing the annealing temperature resulted in a decrease in crystallite size, both for the TiO_2_NT/ZIS_6_ and TiO_2_NT/ZIS_12_ series. The decrease was slightly higher for layers obtained in a 12 h hydrothermal process. This observation suggests that the hydrothermal deposition time plays a more prominent role in influencing the crystal growth kinetics on the TiO_2_NT substrate, potentially due to the unique structural features and enhanced surface area of nanotubular structures.

Figure S2 shows the UV–Vis absorbance spectra, calculated from the reflectance spectrum using the Kubelka Munk equation, of ZnIn_2_S_4_ layers on FTO and TiO_2_NT obtained in 6 h and 12 h hydrothermal process followed by annealing at different temperatures. The vertical lines on the graphs additionally indicate the values of wavelengths below which the absorbance begins to increase for the studied materials. Except for the layers annealed at 500 °C, all samples exhibited high absorption in the visible light range. For the as-received materials and these annealed at 300 and 400 °C, the onset of absorption can be observed between 550 and 600 nm, and its intensity increases as the wavelength decreases. For layers annealed at 500 °C, the onset of absorption is shifted to smaller wavelengths. The same phenomenon is observed for both FTO and TiO_2_NT substrates as well as for both, 6 and 12 h, synthesis times. The energy band gaps (Tab. S2) of the obtained materials were determined from the Tauc plots shown in Fig. S3. The energy band gap of the layers annealed at 300 °C and without annealing are similar, and equal ~ 2.50 eV. In all cases, the material obtained after annealing at 400 °C exhibits the smallest energy band gap. On the other hand, annealing at 500 °C significantly increases the energy gap even up to about 3 eV for the layers obtained in a 6 h synthesis process on TiO_2_NT (Fig. S3b). Increasing of the Energy band gap is likely related to the thermal degradation of the material. This is in agreement with XRD results, which indicated the formation of In_2_O_3_ during annealing at 500 °C. The layers seem to lose their activity in visible light.

**Figure 4:**
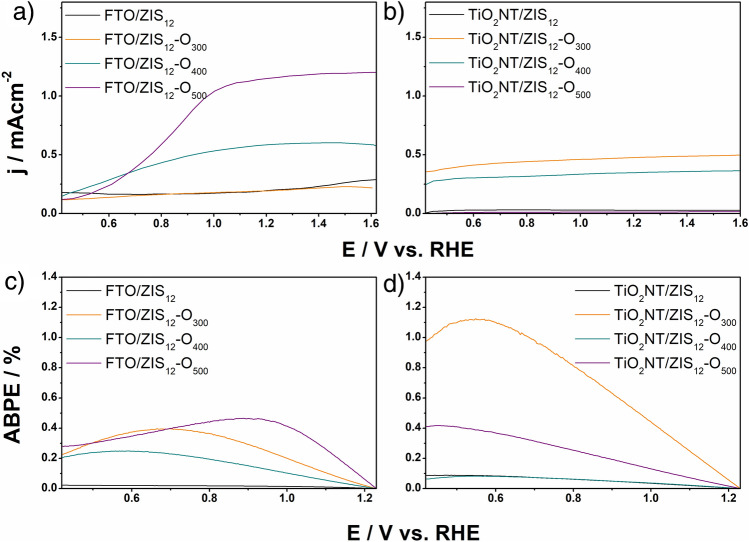
LSV curves of (**a**) FTO/ZIS_12_-based and (**b**) TiO_2_NT/ZIS_12_-based photoanodes; ABPE curves of (**c**) FTO/ZIS_12_-based and (**d**) TiO_2_NT/ZIS_12_-based photoanodes in 0.5 M Na_2_SO_4_.

After the characterization of materials using solid state techniques, the obtained materials were studied as photoanodes for photoelectrochemical water splitting. Figure S4 shows the LSV curve of the FTO/ZIS_6_ electrode recorded without illumination towards cathodic potential. Below − 0.2 V vs. RHE, a sharp increase in cathodic current density can be observed, which indicates the reduction of electrode material. However, at about 0.2 V vs, RHE, a noticeable change in the color of the material was noted as shown in the inset of Fig. S4a. Figure S4b shows LSV curves recorded during intermittent illumination of the FTO/ZIS_6_ photoanode before and after reduction at a potential of 0.2 V vs. RHE for 2 min. As can be seen, the pretreatment significantly affects the photoelectrochemical performance of the tested photoanode. The anodic hump was registered between 0.4 and 0.6 V vs. RHE, which is probably related to partial oxidation of the material after cathodic polarization. Nevertheless, the measured photocurrent at more anodic potential is lower than for the pristine electrode. Thus, further photoelectrochemical measurements (pH = 7) were conducted in potential ranges above 0.4 V vs. RHE to avoid changes in the material. Figure S5 compares the generated photocurrents for FTO/ZIS_6_ and TiO_2_NT/ZIS_6_-based photoanodes. As shown in Fig. S5a, the photocurrent generated by the FTO/ZIS_6_ reaches 0.24 mA cm^−2^ at 1.62 V vs. RHE which is 6 times higher than for TiO_2_NT/ZIS_6_ photoanode, see Fig. S5b. Doubling the time of the hydrothermal process from 6 to 12 h allowed only a slight increase in the photoactivity of the material obtained on FTO (see Fig. 4a). However, as it is presented in Fig. 4b, in the case of TiO_2_NT/ZIS increasing the time of the reaction did not improve the generated photocurrents. Both TiO_2_NT/ZIS_6_ and TiO_2_NT/ZIS_12_ photoanodes reached photocurrent value of ~ 0.03 mA cm^−2^. As shown in Fig. S5a, annealing of FTO/ZIS_6_, led to a reduction in generated photocurrents over the entire chosen potential range. The annealing of FTO/ZIS_12_ at 400 and 500 °C allows for an increase of photoelectroactivity of photoanodes in comparison with not annealed sample (see Fig. 4a). Annealing of ZnIn_2_S_4_ layers on TiO_2_NT results in different photocurrent behavior than for the FTO-based photoanodes (see Fig. S5b and Fig. 4b). ZnIn_2_S_4_ layers on TiO_2_NT (obtained in both 6 h and 12 h process) annealed at 300 and 400 °C generated significantly higher photocurrent than unannealed ones, while annealing at 500 °C led to a decrease in the photoactivity of the material. All samples annealed at 500 °C except FTO/ZIS_12_-O_500_ generated the lowest or near-lowest photocurrents. The use of this temperature in all cases increased the energy gap (see Fig. S3) and decomposition of the material (see Fig. 3). In the case of FTO/ZIS_6_, annealing at 500 °C resulted in a very strong exposure of the substrate surface (see Fig. 1d), which indicates a significant layer quality decrease. Annealing at 500 °C of FTO/ZIS_12_ also resulted in a change in morphology compared to the unannealed material (see Fig. S1d). However, the substrate was not as strongly exposed as in the case of FTO/ZIS_6_-O_500_. As it is shown in Fig. 1h and Fig. S1h ZnIn_2_S_4_ layers on TiO_2_NT annealed at 500 °C do not show significant changes in morphology compared to the non-annealed material. Among the FTO-based photoanodes, FTO/ZIS_12_-O_500_ generates the highest photocurrent density of 1.21 mA cm^−2^ at 1.62 V vs. RHE. The highest photocurrent values using TiO_2_NT as a substrate were achieved for TiO_2_NT/ZIS_12_-O_300_ (0.50 mA cm^−2^ at 1.62 V vs. RHE).

Figure S6 shows Nyquist plots for all the materials obtained. The spectra are typical for porous electrodes^52^. They are seen as straight, almost vertical lines as a characteristic of capacitive behavior. The slightly different shape of spectra was obtained for FTO/ZIS_6_-O_300_. The spectra bends at lower frequencies forming semicircle that may be observed due to the contribution of charge transfer on the interface. There is no simple relation between resistance and annealing temperature for TiO_2_NT/ZIS nor FTO/ZIS electrode materials. FTO/ZIS-based materials annealed at different temperatures exhibited higher series resistance (R_s_) in comparison with bare FTO. It is noteworthy, that for FTO/ZIS_6_ the annealing temperature had a positive impact on the decrease in resistance. In the case of TiO_2_NT/ZIS, the presence of ZnIn_2_S_4_ enhanced the conductivity. The changes in the resistance might be neglected for TiO_2_NT/ZIS synthesized for 12h. It evidences that the substrate electrode affects the electrochemical performance of the electrode material. TiO_2_NT/ZIS electrodes exhibit R_s_ in the range of 4–8 Ω, while for the FTO/ZIS, the R_s_ varies from 25 to 102 Ω.

Applied bias photon-to-current conversion efficiency (ABPE), calculated from the corresponding LSV curves, was the highest for FTO/ZIS_12_-O_500_ (0.46% at 0.93 V vs. RHE) and TiO_2_NT/ZIS_12_-O_300_ (1.12% at 0.55 V vs. RHE) photoanodes, reflecting their superior ability to convert incident photons into photocurrent (see Fig. 4c,d). Importantly, these ABPE results correlate with the observed trend in generated photocurrents for both FTO-based and TiO_2_NT-based photoanodes. Notably, the FTO/ZIS_12_-O_500_ photoanode exhibited higher photocurrents compared to the TiO_2_NT/ZIS_12_-O_300_ photoanode. Despite the FTO-based photoanode generating higher photocurrents, the ABPE value for the TiO_2_NT/ZIS_12_-O_300_ photoanode surpassed that of the FTO/ZIS_12_-O_500_. This suggests that the presence of TiO_2_ in the latter configuration could potentially contribute to improved charge separation dynamics and enhanced kinetics of the water oxidation reaction. In essence, this comparison of ABPE efficiency values between the two photoanodes implies that the incorporation of TiO_2_ in the TiO_2_NT/ZIS_12_-O_300_ configuration has a favorable impact on charge separation and water oxidation kinetics. Although the FTO/ZIS_12_-O_500_ photoanode exhibited higher photocurrents, the ABPE value of the TiO_2_NT/ZIS_12_-O_300_ photoanode signifies a more efficient utilization of the absorbed photons, highlighting the potential advantage of the TiO_2_-based heterojunction in promoting effective charge transfer and water oxidation reactions.

Photoanodes that exhibited the best photoelectrochemical properties in a neutral electrolyte (FTO/ZIS_12_-O_500_ and TiO_2_NT/ZIS_12_-O_300_) were then tested in strongly acidic (0.5 M H_2_SO_4_, pH = 1) and strongly basic (0.5 M NaOH, pH = 14) electrolytes. LSV curves without illumination in acidic, neutral, and alkaline environments of the FTO/ZIS_6_ photoanode were compared to determine the potential range in which the material is stable depending on pH (see Fig. S7). The widest potential range (0.4–2.4 V vs. RHE) was determined for a neutral electrolyte. In this case, in the cathodic direction, it is not the electrolyte that is the limitation, but the stability of the material itself. In contrast, when using an acidic electrolyte, the system had the narrowest potential range: − 0.3 to 0.3 V vs. RHE. On the cathodic side, the main limitation of the acid electrolyte may be the reduction of H^+^ ions. However, on the anodic side, the sharp increase in current density at about 0.3 V vs. RHE is not easy to explain. Using 0.5 M NaOH as an electrolyte, the reduction potential is similar to that obtained in a neutral environment, but the potential range compared to a neutral environment is narrower. The neutral electrolyte proved to be the most suitable in terms of the width of the range of material stability.

**Figure 5:**
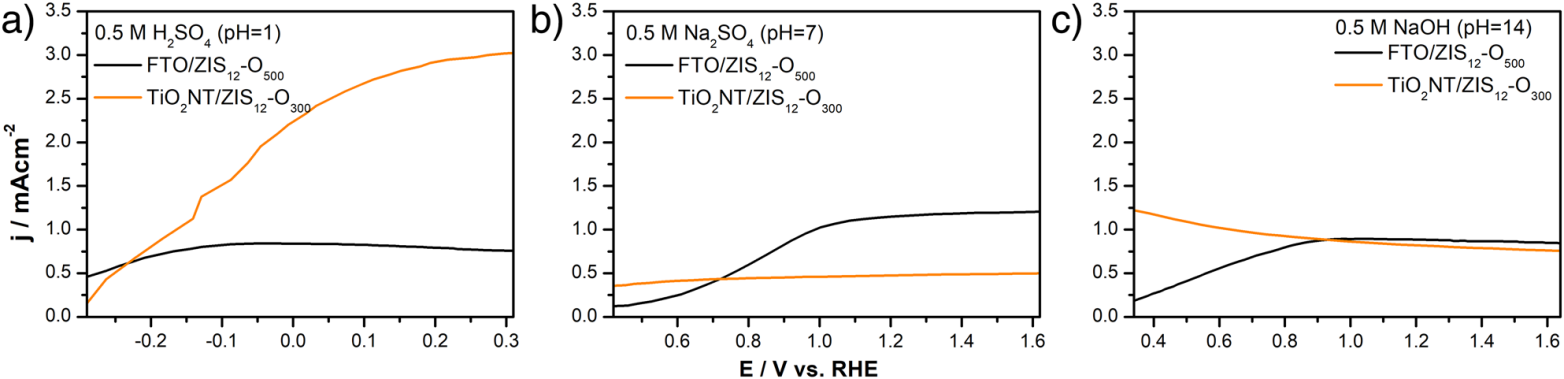
LSV curves of FTO/ZIS_12_-O_300_ and TiO_2_NT/ZIS_12_-O_300_ photoanodes recorded during illumination in (**a**) 0.5 M H_2_SO_4_, (**b**) 0.5 M Na_2_SO_4_ and (**c**) 0.5 M NaOH as electrolytes.

As shown in Fig. 5, the photoelectrochemical properties of FTO/ZIS_12_-O_500_ and TiO_2_NT/ZIS_12_-O_300_ photoanodes in strongly acidic and alkaline conditions were investigated in the previously determined potential ranges (Fig. S7). The use of different pH of the electrolyte affected the values of photocurrents generated by both photoanodes. The effect of pH was more significant for the TiO_2_NT/ZIS_12_-O_300_ photoanode. The FTO/ZIS_12_-O_500_ photoanode generated the highest photocurrent in the neutral electrolyte (1.21 mA cm^−2^ at 1.62 V vs. RHE). In contrast, the use of a strongly acidic electrolyte resulted in a significant increase in the photocurrent values generated by TiO_2_NT/ZIS_12_-O_300_ compared to the other electrolytes. The TiO_2_NT-based photoanode generated photocurrents of 3.02 mA cm^−2^ at 0.31 V vs. RHE in a pH of 1. The photoelectrochemical performance in the acidic, neutral, and basic electrolytes of selected photoanodes was summarized in Table S3.

**Figure 6:**
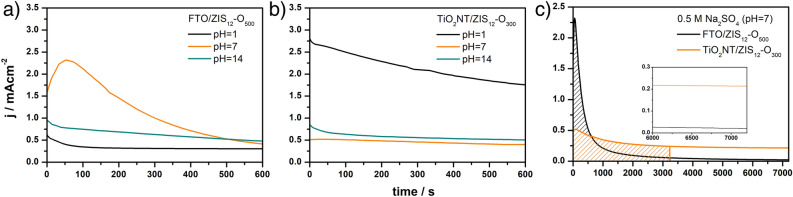
CA curves recorded during illumination of (**a**) FTO/ZIS_12_-O_500_ and (**b**) TiO_2_NT/ZIS_12_-O_300_ photoanodes in different electrolytes, (**c**) CA curves of FTO/ZIS_12_-O_500_ and TiO_2_NT/ZIS_12_-O_300_ recorded during illumination in 0.5 M Na_2_SO_4_.

Measurements using the LSV technique do not provide information on the stability of materials. To investigate the stability of FTO/ZIS_12_-O_500_ and TiO_2_NT/ZIS_12_-O_300_ photoanodes, chronoamperometric measurements were performed. Figure 6a,b show a comparison of the stability of the FTO-based and TiO_2_NT-based photoanodes in strongly acidic (0.5 M H_2_SO_4_, pH = 1), neutral (0.5 M Na_2_SO_4_. pH = 7), and strongly basic (0.5 M NaOH, pH = 14) conditions during exposure for 600 s. From the LSV curves recorded during illumination (Fig. 5), the potentials at which the photocurrents generated by each photoanode were the highest in different media were determined. The FTO-based photoanode was tested at potentials 0.31, 1.62, and 1.64 V vs. RHE in acidic, neutral, and basic electrolytes, respectively. Whereas, the stability of TiO_2_NT-based photoanode was tested at 0.31, 1.62, and 0.34 V vs RHE in acidic, neutral, and basic electrolytes, respectively. The FTO/ZIS_12_-O_500_ photoanode initially generated the highest photocurrents at a pH of 7. However, despite the increase in photocurrents for the first 60 s in a neutral environment, their values began to decrease after that time, and after 600 s the photocurrents generated were 25% of the initial value. In an alkaline environment, the FTO/ZIS_12_-O_500_ photoanode showed better stability, as the photocurrent values dropped by 50% within 600 s, and the drop was not as rapid as in the other electrolytes excluding the first 40 s of the test. The use of an acidic electrolyte resulted in a reduction in the value of generated photocurrents compared to other electrolytes. However, the stability was similar to the results obtained at a pH of 14 (52% decrease in photocurrent values within 600 s of exposure).

As shown in Fig. 6b, the TiO_2_NT-based photoanode initially generated the highest photocurrents in an acidic electrolyte, but their values dropped faster than in the neutral electrolyte, dropping by 37% within 600 s of testing. Whereas under neutral pH this decrease was 21%. The photocurrents generated in 0.5 M NaOH as electrolyte after 600 s of exposure were 59% of the initial values.

The effect of pH on the photoelectrochemical performance of photoanodes was different depending on the substrate used. For the FTO-based photoanode, the most suitable electrolyte regarding photocurrent stability was 0.5 M NaOH (pH = 14). In contrast, the TiO_2_NT/ZIS_12_-O_300_ photoanode was most stable in 0.5 M Na_2_SO_4_. However, the use of a strongly acidic electrolyte (0.5 M H_2_SO_4_, pH = 1) resulted in a significant increase in photocurrent values generated by TiO_2_NT/based photoanode, which after 600 s of exposure were still the highest among the results for all electrolytes used.

The TiO_2_NT/ZIS_12_-O_300_ photoanode in all electrolytes had higher stability than the FTO/ZIS_12_-O_500_ photoanode. To illustrate the significant effect of using TiO_2_NT as a substrate on the stability of the material, long-term measurements (2 h) of the photocurrents generated by the FTO/ZIS_12_-O_500_ and TiO_2_NT/ZIS_12_-O_300_ photoanodes were carried out in a neutral medium (see Fig. 6c). The neutral electrolyte was chosen because, according to shorter stability studies, this electrolyte allowed to achieve the system with the highest stability among all those tested (a decrease of only 21% for the TiO_2_NT/ZIS_12_-O_300_ photoanode). Even though FTO/ZIS_12_-O_500_ generated initially more than four times as high photocurrent values as TiO_2_NT/ZIS_12_-O_300_, these values decreased rapidly, reaching after 613 s the value of 0.4 mA cm^−2^ that is equal to photocurrent generated by TiO_2_NT/ZIS_12_-O_300_. In contrast, the values of TiO_2_NT-based photoanode activity do not change significantly. Moreover, after only 53 min, the charges consumed for photoelectrochemical water splitting on both photoanodes became equal. Which, assuming the same faradaic efficiency, would mean producing the same amount of hydrogen after that time. A similar junction has already been studied, and it was proven that it allowed an increase in generated photocurrents in comparison with TiO_2_^53^. As can be observed in Fig. 6c, after 2 h illumination photocurrent values for FTO-based photoanode decreased by about 99% to reach 0.02 mA cm^−2^, while for TiO_2_NT-based photoanode decreased only by about 58% (0.21 mA cm^−2^).

To investigate in detail the effect of TiO_2_NT substrate on the properties of ZnIn_2_S_4_ layers of the most stable photoanode (TiO_2_NT/ZIS-O_300_), transmission electron microscopy (TEM) studies were performed. The images obtained revealed the typical layered morphology of ZnIn_2_S_4_ (see Fig. S8a,b). As shown in Fig. S8c, high-resolution TEM (HRTEM) images revealed a solid–solid interface between TiO_2_ and ZnIn_2_S_4_. This was confirmed by the respective crystal planes of both compounds. As can be seen in Fig. S8c, the (102) and (006) planes of ZnIn_2_S_4_^54^ and (101) TiO_2_ in anatase form^55^ are evident. This juxtaposition of crystal planes underscores the intimate contact and well-defined interface between the TiO_2_NT substrate and the ZnIn_2_S_4_ layers. The coexistence of these specific crystal planes reinforces the notion of a robust and coherent interface that likely contributes to efficient charge transfer and enhanced photocatalytic performance.

Figure S9 illustrates the proposed possible mechanisms for electron and hole transfer inside the FTO/ZIS_12_-O_500_ and TiO_2_NT/ZIS_12_-O_300_ photoanodes. The band structure and the size of the energy gap of ZnIn_2_S_4_ and TiO_2_NT were investigated using Mott-Schottky (capacitance calculated at 1000 Hz) plots and the Kubelka–Munk function (see Fig. S10). To calculate these values for ZnIn_2_S_4_, the FTO/ZIS_12_ photoanode was studied, since according to the XRD results (Fig. 3), the layers contained no additional compounds. As can be observed in Fig. 3c, In_2_O_3_ was present on the FTO/ZIS_12_-O_500_ photoanode, which affected the size of the energy gap of the whole photoanode. The positive slope of Mott Schottky plots confirms the n-type conductivity of tested materials. For such types of semiconductors, flat band potential (E_fb_) is close to the conduction band (CB). The flat band was located at 0.30 and − 0.68 V vs. RHE for TiO_2_NT and ZnIn_2_S_4_, respectively**.** As expected, the estimated values of E_fb_ are slightly shifted towards the anodic direction in comparison to the reduction of electrode materials. Thus, the Mott-Schottky analysis is in good agreement with CV plots. In the case of the n-type photoelectrodes, the valence band potential is more anodic than water oxidation, thus the valence band (VB) cannot be simply estimated in aqueous electrolytes from electrochemical measurements. The calculated energy gap size for TiO_2_ nanotubes was 3.23 eV, and for ZnIn_2_S_4_ it was 2.35 eV. The location of the conduction band and the size of the energy gap of In_2_O_3_ were taken from the literature^56^. When irradiated with visible light, ZnIn_2_S_4_ generates electron–hole pairs. The presence of In_2_O_3_ (Fig. 3c), which is formed when ZnIn_2_S_4_ layers are annealed at 500 °C, enables the formation of a heterojunction between ZnIn_2_S_4_ and In_2_O_3_. The formed heterojunction in the FTO/ZIS_12_-O_500_ photoanode promotes the transfer of photogenerated electrons to CB of In_2_O_3_, facilitating charge separation. In the case of the TiO_2_NT/ZIS_12_-O_300_ photoanode, according to XRD results (Fig. 3d), we do not observe the presence of In_2_O_3_. However, the use of TiO_2_ nanotubes makes it possible to create a heterojunction between TiO_2_ and ZnIn_2_S_4_. In this case, photoexcited electrons are directed to the CB of TiO_2_, which facilitates charge separation and reduces their recombination rate.

Figure S11 shows SEM images of TiO_2_NT/ZIS_12_ and TiO_2_NT/ZIS_12_-O_300_ after removal of the active layer and an SEM image of pure TiO_2_ nanotubes for comparison. The layers were removed using an ultrasound bath (30 min, in water). As shown in Fig. S11c, the ZIS layer after annealing at 300 °C is difficult to remove compared to the non-annealed layer. Moreover, the layers on the FTO were easy to remove completely, even after annealing. Thus, annealing the layers not only caused changes in morphology and crystal structure but also affected the contact between TiO_2_NT and ZnIn_2_S_4_ facilitating the charge transfer between the components. This was another factor that caused the TiO_2_NT/ZIS_12_-O_300_ photoanode to generate higher photocurrent values in comparison with TiO_2_NT/ZIS_12_. In addition, the deposition of ZnIn_2_S_4_ on TiO_2_ nanotubes and annealing of the layers did not change the morphology of the substrate.

To investigate the reason for the decrease in photoactivity, the characterization of electrode materials was performed after 2h of chronoamperometric measurements. Figure S12 compares the XRD patterns of FTO/ZIS_12_-O_500_ and TiO_2_NT/ZIS_12_-O_300_ photoanodes before and after 2 h of illumination. In the case of the FTO-based photoanode, no significant changes can be observed. The XRD pattern of the TiO_2_NT/ZIS_12_-O_300_ photoanode after 2 h of exposure showed only an increase in the peak corresponding to the (006) plane (~ 21.5°). Despite this change in the case of the TiO_2_NT-based photoanode, it can be concluded that in terms of composition, the material is stable regardless of the substrate used. Thus, it is not the crystallinity of ZIS that has an impact on the degradation of the material during long-term illumination.

Cross-section SEM images of FTO/ZIS_12_, FTO/ZIS_12_-O_500_, TiO_2_TN/ZIS_12_, and TiO_2_TN/ZIS_12_-O_300_ photoanodes before and after 2 h of illumination (see Fig. S13) allowed to estimate the thickness of the obtained ZnIn_2_S_4_. Cross-sectional samples were obtained by carefully preparing the coated substrates to expose the inner layers for observation under the SEM. Using SEM could be visualized the cross-section of the ZnIn_2_S_4_-coated layers on both substrates. By analyzing SEM images, the thickness of the deposited ZnIn_2_S_4_ layer was measured at various points on the substrates. The thickness of ZnIn_2_S_4_ layers was not uniform throughout the whole surface on both FTO and TiO_2_NT. The measured thicknesses were in the range of 3–12 μm. There was no clear evidence that annealing affects the thickness of the deposited layers. However, the use of the hydrothermal process resulted in layers of different thicknesses within the same electrode. It was also not possible to determine the significant changes in layer thickness during long-term exposure. Thus, with the use of SEM, it was not possible to observe changes in photoanodes that negatively affected their performance during exposure.

**Figure 7:**
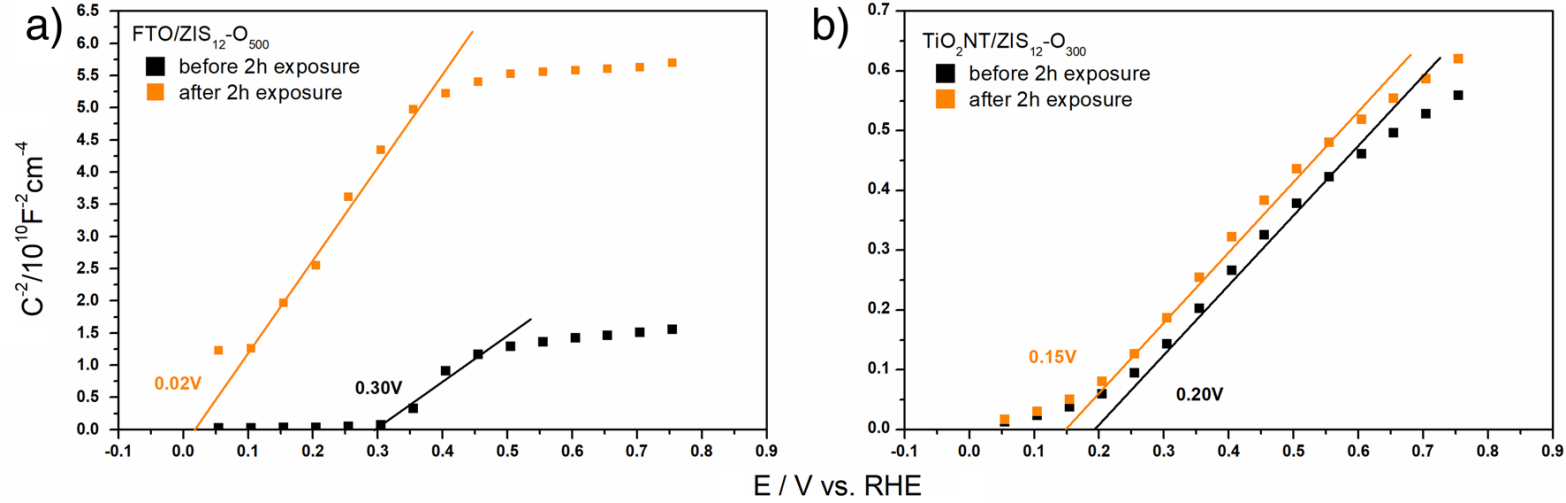
Mott–Schottky plots of (**a**) FTO/ZIS_12_-O_500_ and (**b**) TiO_2_NT/ZIS_12_-O_300_ before and after 2h illumination with simulated sunlight.

Flat band potentials for photoanodes before and after 2h of exposure were determined using Mott Schottky analysis (Fig. 7). The flat band potential of FTO/ZIS_12_-O_500_ was shifted from 0.3 V to 0.02 V vs. RHE after 2 h exposure. In the case of the TiO_2_NT/ZIS_12_-O_500_ photoanode, the shift of the flat band potential was only 0.05 V. For both substrates, a cathodic shift in the flat band potential of the materials was observed. It evidences that the presence of the substrate affects the electrochemical response of the photoanode. Despite the theoretically favorable shift in the case of FTO, it indicates lower stability of the FTO-based photoanode and more changes in the material during illumination.

**Figure 8:**
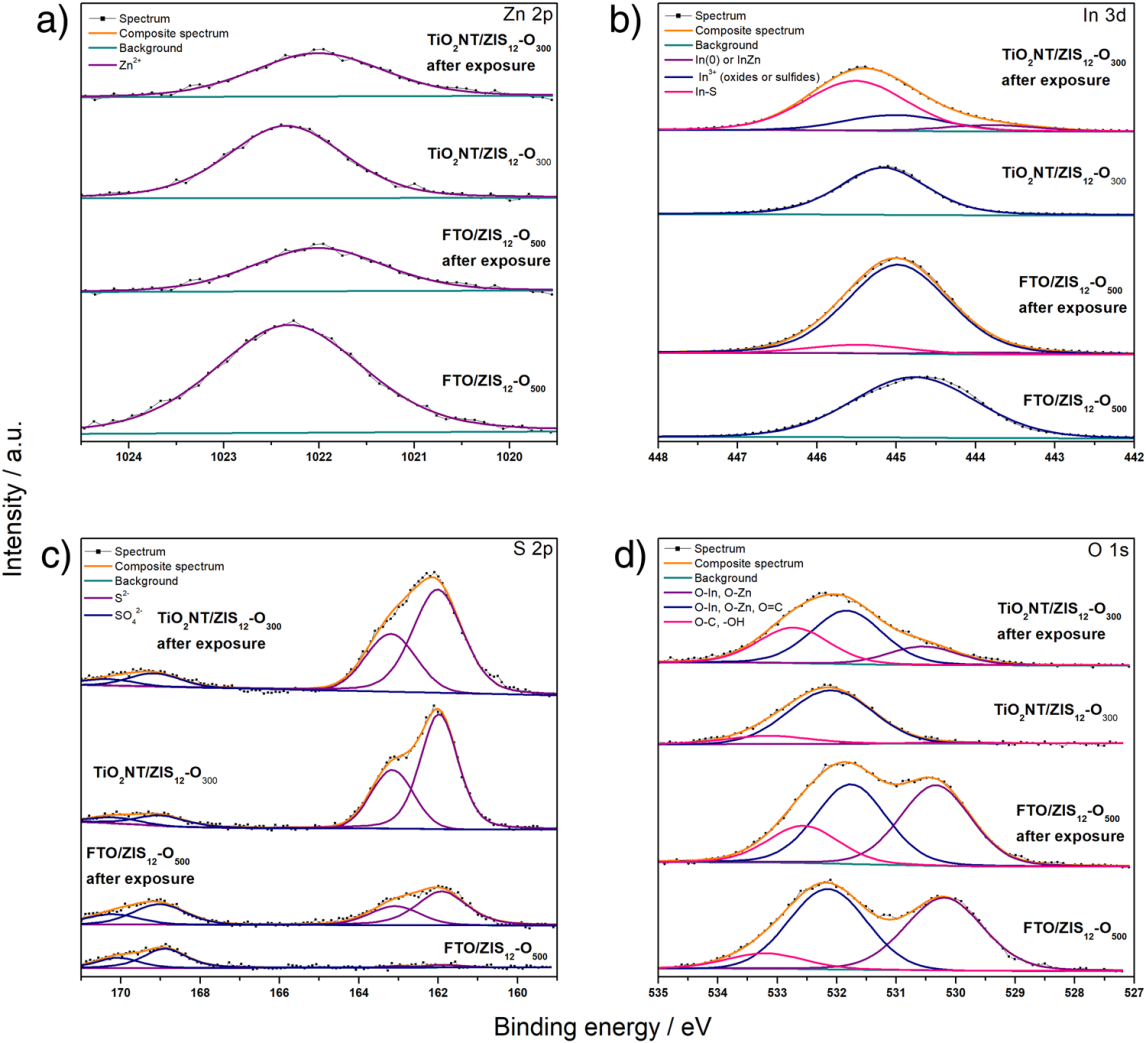
High resolution XPS spectrum of (**a**) Zn 2p, (**b**) In 3d, (**c**) S 2p and (**d**) O 1s of FTO/ZIS_12_-O_500_ and TiO_2_NT/ZIS_12_-O_300_ before and after 2 h of exposure to simulated sunlight.

X-ray photoelectron spectroscopy (XPS) was carried out for the FTO/ZIS_12_-O_500_ and TiO_2_NT/ZIS_12_ O_300_ photoanodes before and after 2 h of exposure to simulated sunlight. The XPS measurement was performed to determine the changes in the surface composition and to evaluate the oxidation level of the elements. Figure 8 shows the high-resolution XPS spectra of the Zn 2p, In 3d, S 2p, and O 1s regions. The high-resolution Zn 2p_3/2_ spectrum, shown in Fig. 8a, was fitted with a single line lying at an energy of about 1022.2 eV, which indicates the presence of zinc (II)^39,56^ on both FTO and TiO_2_NT before exposure. The post-exposure spectra have a lower intensity and are shifted toward a lower energy of 1022.0 eV. This shift may be related to a change in the stoichiometry of the ZIS compound after illumination. Such a shift in the case of zinc may indicate a decrease in the proportion of zinc bound to sulfur instead of oxygen (to an element with higher electronegativity)^58^. Figure 8b shows the spectrum of photoanodes in the indium region. For samples before exposure, the spectrum in the In 3d_5/2_ region shows a single line at 444.8 eV for FTO/ZIS_12_-O_500_ and 445.0 eV for TiO_2_NT/ZIS_12_-O_300_ indicating the presence of In^3+^ in sulfide and oxide/sulfide, respectively^58,59^. For the TiO_2_NT/ZIS_12_-O_300_ photoanode after exposure, the spectrum in the In 3d_5/2_ region was fitted with three lines with a binding energy of 443.8 eV, 445.0 eV, and 445.5 eV indicating the presence of metallic indium, indium oxide and indium sulfide, respectively^58,59^. In the case of FTO/ZIS_12_-O_500_ after exposure, only two maxima were obtained. The position of peaks was the same as for TiO_2_NT/ZIS_12_-O_300_ material confirming the presence of In-O and In-S bonds. It is noteworthy that after the illumination the intensity of the signal attributed to indium sulfide increases. It is especially visible for TiO_2_NT/ZIS_12_-O_300_. It might be related to the removal of the passive layer formed on the electrode material during the annealing process. The spectrum in the S 2p region of the photoanodes was fitted by two doublets, see Fig. 8c. The first main 2p_3/2_ line at 161.8 eV indicates the presence of sulfur in the sulfides^58^ and the second 2p_3/2_ line at 168.9 eV indicating the presence of sulfates (VI)^61^. The most significant difference between the spectra in the sulfur region of photoanodes before exposure is the intensity of the lines corresponding to S^2−^. In the case of TiO_2_NT/ZIS_12_-O_300,_ it is significantly higher than that for FTO/ZIS_12_-O_500_. This difference is related to the formation of an indium oxide layer on the surface of the electrode during annealing at 500 °C. It is consistent with the XRD results showing that in the materials annealed at 500 °C indium oxide forms whereas in the case of TiO_2_NT/ZIS_12_-O_300_ annealed at 300 °C the indium oxide layer was not formed.

The S 2p_3/2_ spectrum of TiO_2_NT/ZIS_12_-O_300_ did not change significantly after exposure. For FTO/ZIS_12_-O_500_, a significant increase in the proportion of sulfur in the form of S^−2^ can be seen, with no change in the intensity of the line representing sulfates. It confirms, that illumination affects the composition of material and leads to changes in the stoichiometry between S and O on the surface.

The spectrum in the oxygen region shown in Fig. 8d was fitted with three lines. The first signal at ~ 530.1 eV indicates the presence of metal oxides. The second maximum at ~ 532.1 eV indicates the presence of non-stoichiometric metal oxides and/or sulfates in both materials before illumination. After illumination, this maximum is slightly shifted toward a lower binding energy of 531.9 eV. It is related to the adsorption of water molecules from electrolyte, followed by a change of stoichiometry ratio between S and O atoms affecting binding energy in the Me-S(O) system. The presence of water in photoanodes before and after exposure is confirmed by the presence of maxima at 533.2 eV and 532.7, respectively^39^.

## Summary

Our study marks a significant achievement as we successfully synthesized ZnIn_2_S_4_ layers on TiO_2_ nanotubes for the first time. The annealing process performed under varying air temperatures exerted effects on the crystallographic structure, substrate adhesion, and composition of these layers, leading to consequential alterations in the resulting photocurrent values. Importantly, the impact of annealing exhibited substrate-specific variations, reflecting the intricate interplay between material properties and thermal treatment.

Through optimization, our efforts culminated in the creation of an optimized TiO_2_NT-based photoanode, showcasing a remarkable enhancement in photocurrent density, reaching 0.49 mA cm^−2^ at 0.8 V vs. Ag/AgCl (3 M KCl). This optimized configuration demonstrated a significant advantage over photoanodes fabricated using FTO glass substrates, primarily in terms of stability during illumination. Despite an initial lower photoelectrochemical activity, the TiO_2_NT-based photoanode exhibited a pronounced capability for efficient hydrogen generation through photoelectrochemical water splitting. After just 53 min of illumination, it outperformed its FTO-based counterparts. The stark contrast became even more evident after 2 h, as FTO-based photoanodes suffered a precipitous 99% decrease in photocurrent values, while TiO_2_NT-based counterparts experienced a notably milder 58% decrease. This resilience, attributed to the formed heterojunction at the ZnIn_2_S_4_-TiO_2_NT interface, underscores the potential for sustained high photocurrent values over extended periods. In addition to the aforementioned findings, we expanded our investigation by systematically exploring the influence of pH on the performance of the photoanodes. As observed, the pH-dependent measurements provided critical insights into the response of the TiO_2_NT and FTO-based ZnIn_2_S_4_ photoanode under varying chemical environments. These measurements enabled a comprehensive understanding of the photoelectrochemical behavior and stability of the photoanode across acidic, neutral, and basic conditions.

Thus, the use of TiO_2_NT as a substrate for the deposition of other photoanode materials could be a promising way to maximize their working time, which is a very important factor in allowing them to be used on a larger scale.

### Supplementary Information

Supplementary Information.

## Data Availability

The datasets generated and/or analysed during the current study are available in the BRIDGE OF KNOWLEDGE repository (https://mostwiedzy.pl/pl/open-research-data/x-ray-diffraction-of-znin2s4-layers-on-tio2nt-and-fto-annealed-at-different-temperatures, 41301083143118-0).

## References

[1] Kang D (2015). Electrochemical synthesis of photoelectrodes and catalysts for use in solar water splitting. Chem. Rev..

[2] Navarro Yerga, R. M., Alvarez-Galvan, M. C., Vaquero, F., Arenales, J. & Fierro, J. L. G. Hydrogen production from water splitting in *Renewable hydrogen technologies*, 43–61. 10.1016/B978-0-444-56352-1.00003-9 (Elsevier Science, 2013).

[3] Ye KH (2019). Enhancing photoelectrochemical water splitting by combining work function tuning and heterojunction engineering. Nat. Commun..

[4] Chen X, Shen S, Guo L, Mao SS (2010). Semiconductor-based photocatalytic hydrogen generation. Chem. Rev..

[5] Fujishima A, Honda K (1972). Electrochemical photolysis of water at a semiconductor electrode. Nature.

[6] Jelinska A (2018). Enhanced photocatalytic water splitting on very thin WO_3_ films activated by high-temperature annealing. ACS Catal..

[7] Rani BJ (2019). WO_3_ nanocubes for photoelectrochemical water-splitting applications. J. Phys. Chem. Solids.

[8] Makimizu Y (2019). Effects of low oxygen annealing on the photoelectrochemical water splitting properties of α-Fe_2_O_3_. J. Mater. Chem. A.

[9] Tokubuchi T (2021). Enhanced photoelectrochemical water splitting efficiency of hematite (α-Fe_2_O_3_)-Based photoelectrode by the introduction of maghemite (γ-Fe_2_O_3_) nanoparticles. J. Photochem. Photobiol. A Chem..

[10] Siavash Moakhar R (2021). Photoelectrochemical water-splitting using CuO-based electrodes for hydrogen production: A review. Adv. Mater..

[11] Li J (2019). Copper oxide nanowires for efficient photoelectrochemical water splitting. Appl. Catal. B Environ..

[12] Sun, X, Li, Q., Jiang, J. & Mao, Y. Morphology-tunable synthesis of ZnO nanoforest and its photoelectrochemical performance. *Nanoscale* **6**, 8769–8780. 10.1039/c4nr01146e (2014). 10.1039/c4nr01146e 24954305

[13] Canela, M. C., Alberici, R. M. & Jardim, W. F. Gas-phase destruction of H_2_S using TiO_2_ / UV-VIS. *J. Photochem. Photobiol., A* **112**, 73–80. 10.1016/S1010-6030(97)00261-X (1998).

[14] Riente P, Noël T (2019). Application of metal oxide semiconductors in light-driven organic transformations. Catal. Sci. Technol..

[15] DeAngelis AD, Kemp KC, Gaillard N, Kim KS (2016). Antimony(III) sulfide thin films as a photoanode material in photocatalytic water splitting. ACS Appl. Mater. Interfaces.

[16] Rasool S (2019). Effect of annealing on the physical properties of thermally evaporated In_2_S_3_ thin films. Curr. Appl. Phys..

[17] Song JP, Yin PF, Mao J, Qiao SZ, Du XW (2017). Catalytically active and chemically inert CdIn_2_S_4_ coating on a CdS photoanode for efficient and stable water splitting. Nanoscale.

[18] Sinsermsuksakul P (2014). Overcoming efficiency limitations of SnS-based solar cells. Adv. Energy Mater..

[19] Tedstone AA, Lewis DJ, O’Brien P (2016). Synthesis, properties, and applications of transition metal-doped layered transition metal dichalcogenides. Chem. Mater..

[20] Yan C (2017). Space-confined chemical vapor deposition synthesis of ultrathin HfS_2_ flakes for optoelectronic application. Adv. Funct. Mater..

[21] Li Y, Hu Y, Peng S, Lu G, Li S (2009). Synthesis of CdS nanorods by an ethylenediamine assisted hydrothermal method for photocatalytic hydrogen evolution. J. Phys. Chem. C.

[22] Tang Y, Hu X, Liu C (2014). Perfect inhibition of CdS photocorrosion by graphene sheltering engineering on TiO_2_ nanotube array for highly stable photocatalytic activity. Phys. Chem. Chem. Phys..

[23] Cao S (2016). Band alignment engineering for improved performance and stability of ZnFe_2_O_4_ modified CdS/ZnO nanostructured photoanode for PEC water splitting. Nano Energy.

[24] Cheng L, Xiang Q, Liao Y, Zhang H (2018). CdS-based photocatalysts. Energy Environ. Sci..

[25] Chaudhari NS (2011). Ecofriendly hydrogen production from abundant hydrogen sulfide using solar light-driven hierarchical nanostructured ZnIn_2_S_4_ photocatalyst. Green Chem..

[26] Wang H (2020). Highly active deficient ternary sulfide photoanode for photoelectrochemical water splitting. Nat. Commun..

[27] Ma D (2018). Multiple carrier-transfer pathways in a flower-like In_2_S_3_/CdIn_2_S_4_/In_2_O_3_ ternary heterostructure for enhanced photocatalytic hydrogen production. Nanoscale.

[28] Juine RN, Sahu BK, Das A (2021). Recyclable ZnS QDs as an efficient photocatalyst for dye degradation under the UV and visible light. New J. Chem..

[29] Kempken B (2015). Synthesis, optical properties, and photochemical activity of zinc-indium-sulfide nanoplates. RSC Adv..

[30] Janani R, Sahoo MK, Gupta B, Rao GR, Singh S (2019). Multifunctional hierarchical ZnIn_2_S_4±δ_ microflowers with photocatalytic and pseudocapacitive behavior. Sol. Energy.

[31] Sun B (2021). O, S-dual-vacancy defects mediated efficient charge separation in ZnIn_2_S_4_/Black TiO_2_ heterojunction hollow spheres for boosting photocatalytic hydrogen production. ACS Appl. Mater. Interfaces.

[32] Gunjal AR (2020). A hierarchical SnS@ZnIn_2_S_4_ marigold flower-like 2D nano-heterostructure as an efficient photocatalyst for sunlight-driven hydrogen generation. Nanoscale Adv..

[33] Wang S, Guan BY, Lou XWD (2018). Construction of ZnIn_2_S_4_-In_2_O_3_ hierarchical tubular heterostructures for efficient CO_2_ photoreduction. J. Am. Chem. Soc..

[34] Su L, Ye X, Meng S, Fu X, Chen S (2016). Effect of different solvent on the photocatalytic activity of ZnIn_2_S_4_ for selective oxidation of aromatic alcohols to aromatic aldehydes under visible light irradiation. Appl. Surf. Sci..

[35] Peng X (2020). Nanohybrid photocatalysts with ZnIn_2_S_4_ nanosheets encapsulated UiO-66 octahedral nanoparticles for visible-light-driven hydrogen generation. Appl. Catal. B Environ..

[36] Cavdar O (2023). Photocatalytic hydrogen evolution from glycerol-water mixture under visible light over zinc indium sulfide (ZnIn_2_S_4_) nanosheets grown on bismuth oxychloride (BiOCl) microplates. J. Colloid Interface Sci..

[37] Meng L (2018). Simultaneous manipulation of O-doping and metal vacancy in atomically thin Zn_10_In_16_S_34_ nanosheet arrays toward improved photoelectrochemical performance. Angew. Chemie - Int. Ed..

[38] Xu W, Gao W, Meng L, Tian W, Li L (2021). Incorporation of sulfate anions and sulfur vacancies in ZnIn_2_S_4_ photoanode for enhanced photoelectrochemical water splitting. Adv. Energy Mater..

[39] Peng S, Zhu P, Thavasi V, Mhaisalkar SG, Ramakrishna S (2011). Facile solution deposition of ZnIn_2_S_4_ nanosheet films on FTO substrates for photoelectric application. Nanoscale.

[40] Sima C, Grigoriu C, Antohe S (2010). Comparison of the dye-sensitized solar cells performances based on transparent conductive ITO and FTO. Thin Solid Films.

[41] Mou J (2023). A highly efficient visible-light-driven photocatalytic fuel cell with ZnIn_2_S_4_/PANI/TiO_2_/Ti photoanode for simultaneous rhodamine B degradation and electricity generation. New J. Chem..

[42] Mahadik MA, Shinde PS, Cho M, Jang JS (2016). Metal oxide top layer as an interfacial promoter on a ZnIn_2_S_4_/TiO_2_ heterostructure photoanode for enhanced photoelectrochemical performance. Appl. Catal. B Environ..

[43] Zhu Q (2020). Rational design of 3D/2D In_2_O_3_ nanocube/ZnIn_2_S_4_ nanosheet heterojunction photocatalyst with large-area ‘high-speed channels’ for photocatalytic oxidation of 2,4-dichlorophenol under visible light. J. Hazard. Mater..

[44] Shi X (2020). WO_3_/ZnIn_2_S_4_ heterojunction photoanodes grafting silane molecule for efficient photoelectrochemical water splitting. Electrochim. Acta.

[45] Ali I, Suhail M, Alothman AA, Alwarthan A (2018). Recent advances in syntheses, properties and applications of TiO_2_ nanostructures. RSC Adv..

[46] Lee K, Mazare A, Schmuki P (2014). One-dimensional titanium dioxide nanomaterials: Nanotubes. Chem. Rev..

[47] Reyes-Gil KR, Robinson DB (2013). WO_3_-enhanced TiO_2_ nanotube photoanodes for solar water splitting with simultaneous wastewater treatment. ACS Appl. Mater. Interfaces.

[48] Koiki BA (2020). Cu_2_O on anodised TiO_2_ nanotube arrays: A heterojunction photoanode for visible light assisted electrochemical degradation of pharmaceuticals in water. Electrochim. Acta.

[49] Trzciński K, Szkoda M, Siuzdak K, Sawczak M, Lisowska-Oleksiak A (2016). Electrochemical and photoelectrochemical characterization of photoanodes based on titania nanotubes modified by a BiVO_4_ thin film and gold nanoparticles. Electrochim. Acta.

[50] Chen Z (2009). Low-temperature and template-free synthesis of ZnIn_2_S_4_ microspheres. Inorg. Chem..

[51] Kumar BH, Kumar MCS (2020). On the conversion of amorphous In_2_S_3_ thin films to polycrystalline In_2_S_3_ and to In_2_O_3_ through thermal oxidation process. Mater. Sci. Semicond. Process.

[52] Ariyoshi K (2022). Electrochemical impedance spectroscopy part 1: fundamentals. Electrochemistry.

[53] Liu Q (2014). 2D ZnIn_2_S_4_ Nanosheet/1D TiO_2_ Nanorod heterostructure arrays for improved photoelectrochemical water splitting. ACS Appl. Mater. Interfaces.

[54] Su T (2021). Sulfur vacancy and Ti_3_C_2_T_x_ cocatalyst synergistically boosting interfacial charge transfer in 2D/2D Ti_3_C_2_T_x_/ZnIn_2_S_4_ heterostructure for enhanced photocatalytic hydrogen evolution. Adv. Sci..

[55] Štengl V, Grygar TM (2011). The simplest way to iodine-doped anatase for photocatalysts activated by visible light. Int. J. Photoenergy.

[56] Sharma MD, Mahala C, Basu M (2020). Photoelectrochemical water splitting by In_2_S_3_/In_2_O_3_ composite nanopyramids *ACS Appl*. Nano Mater..

[57] Wagner, A. D. *et al.* NIST Standard Reference Database 20 https://srdata.nist.gov/xps/ (2003).

[58] Moulder JF (1992). Handbook of X-ray Photoelectron Spectroscopy: A Reference Book of Standard Spectra for Identification and Interpretation of XPS Data.

[59] Biesinger MC (2011). Resolving surface chemical states in XPS analysis of first row transition metals, oxides and hydroxides: Cr, Mn, Fe, Co and Ni. Appl. Surf. Sci..

[60] Daraz U, Ansari TM, Arain SA, Mansoow MA, Mazhar M (2020). (2020) Structural, topographical and optoelectronic properties of ZnIn2S4 thin films deposited from dual source using aerosol assisted chemical vapour deposition (AACVD) Technique. Gen. Phys..

[61] Fantauzzi M, Elsener B, Atzei D, Rigoldi A, Rossi A (2015). Exploiting XPS for the identification of sulfides and polysulfides. RSC Adv..

