# Supplementary material for: The new method of ZnIn2S4 synthesis on the titania nanotubes substrate with enhanced stability and photoelectrochemical performance
Source: Sci Rep. 2023 Dec 2;13:21263. doi: 10.1038/s41598-023-48309-9 (PMC10692104; doi:10.1038/s41598-023-48309-9)
Supplement: Supplementary file 1 — Supplementary Information. [file 41598_2023_48309_MOESM1_ESM.docx]

**Experimental**

***Substrate preparation***

The fluorine doped tin oxide coated glass slide (FTO, ~7Ωsq^-1^, Sigma Aldrich) was cleaned using an ultrasonic bath (15 min) in acetone, ethanol, isopropanol and distilled water respectively. Titanium dioxide nanotubes were prepared by one-step electrochemical oxidation (anodization) of titanium foil (0.127 mm thick, annealed, 99%, Alfa Aesar) according to a previously optimized procedure [1]. First, the titanium sheets, placed in a mixture of acetone and isopropanol (volume ratio 1:1), were cleaned using ultrasound for 20 min and then dried in air. Anodization was carried out in a two-electrode system in which both the anode and cathode were titanium sheets. Electrodes were placed in 50 cm^3^ water:glycol mixture (volume ratio 1:19) about 2 cm separated from each other. The electrolyte contained 0.27 M ammonium fluoride and 1 M phosphoric acid (V). The anodization process was carried out for 2 h at the constant voltage of 40 V using a programmable switching D.C. power supply PSB-2400L2, GW Instek. The nanotubes obtained on the titanium foil were washed with a solution of 100 μl HF in 50 cm^3^ water (to remove inorganic impurities), followed by distilled water. In the last step, the obtained material was calcined at 450˚C for 2 h. As a result, TiO_2_NT were transformed from amorphous to crystalline form.

***Preparation of photoanodes***

The precursor solution contained 0.136 g ZnCl_2_, 0.586 g InCl_3_ · 4H_2_O and 0.304 g thiourea in 100 ml deionized water. FTO or TiO_2_NT sheet was placed into the autoclave containing 10 ml precursor solution. The substrate sheet was leaned against the wall of the autoclave at an angle of approximately 45˚. Hydrothermal synthesis was carried out at 160˚C. The process was carried out for 6 or 12 h to obtain ZIS_6_ and ZIS_12_ layers, respectively. The resulting layers were washed in distilled water and ethanol and then dried with air at room temperature.

The photoanodes obtained in the first stage were then annealed in air atmosphere at different temperatures of 300, 400 and 500˚C to obtain ZIS-O_300_, ZIS-O_400_ and ZIS-O_500_ layers, respectively.

***Materials characterization and PEC measurements***

The crystal phase of the obtained materials was characterized by the X-ray diffraction (XRD) method using a Phillips X'Pert Pro diffractometer with Cu Kα radiation (λ = 1.542 Å). Using the Scherrer equation[2], the average crystallite size was calculated:

$$d=\frac{K \times\lambda}{\beta\times cos\theta}$$

where d is the crystallite size [m], λ is the X-ray wavelength [m], β is the width of the peak [radian] (full width at half maximum) and K is the Scherrer constant [-]. The morphology was observed using scanning electron microscopy (SEM, FEIQuanta FEG 250, FEI Company, Hillsboro, OR, USA). TEM studies were carried out using an EMD UL 3 - 4 nm microscope equipped with an electron gun with a LaB_6_ cathode. To determine the surface composition, XPS analysis was carried out with a PHI VersaProbeII instrument (ULVAC-PHI, Chigasaki, Japan) using monochromatic X-ray of the Al Kα line (1486.6 eV). Perkin Elmer Lambda 18 UV – vis spectrometer equipped with a diffuse reflectance accessory was used to determine the optical absorption of obtained photoanodes. UV-vis absorbance spectra, calculated from the reflectance spectrum using the Kubelka Munk equation [3]:

$$F\left( R \right)=\frac{{(1-R)}^{2}}{2R}=\frac{\varepsilon}{s}$$

where R is the reflectance, ε is the molar absorbace coefficient, s is the light scattering coefficient.

Photoelectrochemical performance was tested in 0.5 M H_2_SO_4_ (pH 1), 0.5 M Na_2_SO_4_ (pH 7), and 0.5 NaOH (pH=14) electrolytes, under simulated solar light illumination from a lamp equipped with an AM1.5 filter (LOT LS0500/1). The intensity of light was adjusted to 100 mW cm^-2^ using an Ophir power meter. Photoelectrochemical measurements were carried out in the three-electrode system using PGSTAT204, Metrohm Autolab B.V. Pt mesh as counter electrode and an Ag/AgCl/3.0 M KCl as reference electrode was used. The scanning rate for linear sweep voltammetry (LSV) measurements was 20 mVs^-1^, and for cyclic voltammetry (CV) was 50 mVs^-1^. The measured potentials vs. Ag/AgCl/3M KCl were converted to the reversible hydrogen electrode (RHE) scale according to the Nernst equation:

$$E_{RHE}=E_{Ag/AgCl/3M KCl}+E_{Ag/AgCl/3M KCl}^{0}+0.059pH$$

where E_RHE_ is the converted potential vs. RHE, E_AgAg/Cl_ is the experimentally measured potential against Ag/AgCl/3M KCl reference and E^0^_Ag/AgCl/3M KCl_ = 0.21 V at 25^o^.

Applied bias photon-to-current efficiency (ABPE) of the photoanode was calculated based on the LSV curves according to the following formula [4]:

$$ABPE \left( \% \right)=\left( \frac{I\times\left( 1.23-V_{app} \right)}{P_{incident}} \right)\times100\%$$

where V_app_ is the applied external potential versus RHE, I is the measured current density and P_incident_ is the power density of the incident light (mW cm^-2^).


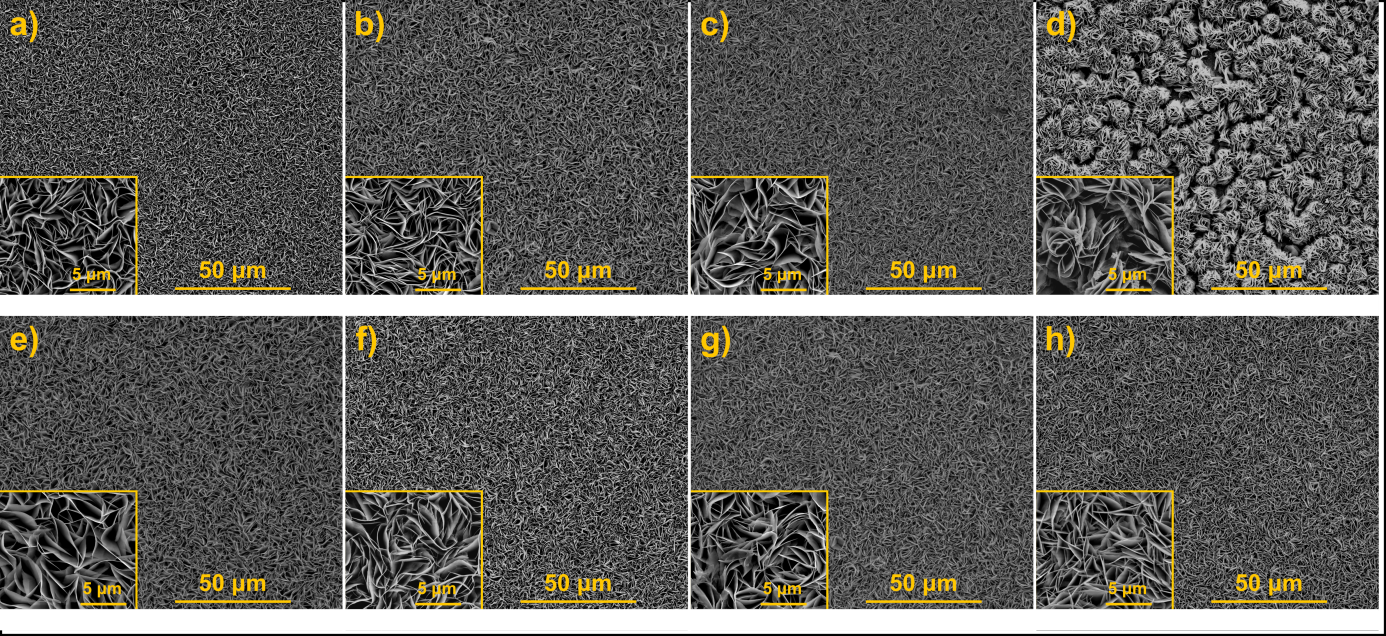


**Fig. S1.** SEM images of a) FTO/ZIS_12_, b) FTO/ZIS_12_-O_300_, c) FTO/ZIS_12_-O_400_, d) FTO/ZIS_12_-O_500_, e) TiO_2_NT/ZIS_12_, f) TiO_2_NT/ZIS_12_-O_300_, g) TiO_2_NT/ZIS_12_-O_400_ and h) TiO_2_NT/ZIS_12_-O_500_.

**Table S1.** Average crystal size of ZnIn_2_S_4_ (62.5^o^) of photoanodes.

| Material | Average crystal size [nm] |
| --- | --- |
| FTO/ZIS_6_ | 42.94 |
| FTO/ZIS_6_-O_300_ | 42.49 |
| FTO/ZIS_6_-O_400_ | 44.22 |
| FTO/ZIS_6_-O_500_ | 42.38 |
| FTO/ZIS_12_ | 41.49 |
| FTO/ZIS_12_-O_300_ | 41.17 |
| FTO/ZIS_12_-O_400_ | 42.60 |
| FTO/ZIS_12_-O_500_ | 43.52 |
| TiO_2_NT/ZIS_6_ | 102.30 |
| TiO_2_NT/ZIS_6_-O_300_ | 96.92 |
| TiO_2_NT/ZIS_6_-O_400_ | 83.05 |
| TiO_2_NT/ZIS_6_-O_500_ | 65.34 |
| TiO_2_NT/ZIS_12_ | 132.98 |
| TiO_2_NT/ZIS_12_-O_300_ | 125.65 |
| TiO_2_NT/ZIS_12_-O_400_ | 92.20 |
| TiO_2_NT/ZIS_12_-O_500_ | 89.50 |





**Fig. S2.** UV–Vis absorbance spectra of a) FTO/ZIS_6_, b) TiO_2_NT/ZIS_6_, c) FTO/ZIS_12_ and d) TiO_2_NT/ZIS_12_ photoanodes before and after annealing at 300, 400 and 500˚C.





**Fig. S3.** Resulting from UV-vis spectra Tauc plots of a) FTO/ZIS_6_, b) TiO_2_NT/ZIS_6_, c) FTO/ZIS_12_ and d) TiO_2_NT/ZIS_12_ photoanodes before and after annealing at 300, 400 and 500˚C.

**Table S2.** Energy band gaps of the obtained materials determined from the Tauc plots.

| Material | Energy band gap [eV] | Wavelength equivalent to the energy band gap [nm] |
| --- | --- | --- |
| FTO/ZIS_6_ | 2.47 | 502.83 |
| FTO/ZIS_6_-O_300_ | 2.50 | 496.80 |
| FTO/ZIS_6_-O_400_ | 2.18 | 569.72 |
| FTO/ZIS_6_-O_500_ | 2.78 | 446.763 |
| FTO/ZIS_12_ | 2.37 | 524.05 |
| FTO/ZIS_12_-O_300_ | 2.32 | 535.34 |
| FTO/ZIS_12_-O_400_ | 2.25 | 552.00 |
| FTO/ZIS_12_-O_500_ | 2.82 | 440.43 |
| TiO_2_NT/ZIS_6_ | 2.36 | 526.27 |
| TiO_2_NT/ZIS_6_-O_300_ | 2.34 | 530.77 |
| TiO_2_NT/ZIS_6_-O_400_ | 2.26 | 549.56 |
| TiO_2_NT/ZIS_6_-O_500_ | 3.06 | 405.88 |
| TiO_2_NT/ZIS_12_ | 2.45 | 506.94 |
| TiO_2_NT/ZIS_12_-O_300_ | 2.34 | 530.77 |
| TiO_2_NT/ZIS_12_-O_400_ | 4.70 | 264.26 |
| TiO_2_NT/ZIS_12_-O_500_ | 2.75 | 451.64 |


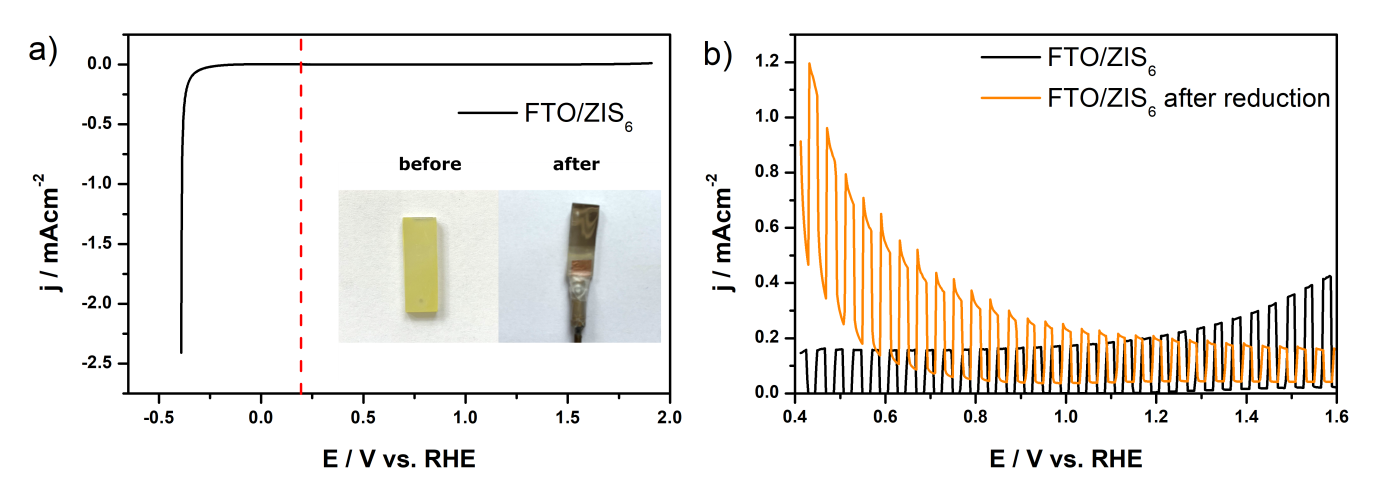


**Fig. S4.** LSV curve of FTO/ZIS_6_, b) LSV curves of FTO/ZIS_6_ before and after reduction (E=-0.6 V, time: 2min), inset: photos of FTO/ZIS_6_ before and after reduction (E=-0.6 V, time: 2min).





**Fig. S5.** LSV curves of a) FTO/ZIS_12_-based and b) TiO_2_NT/ZIS_12_-based photoanodes in 0.5 M Na_2_SO_4_.





**Fig. S6.** EIS Nyquist plots of a) FTO/ZIS_6_, b) TiO_2_NT/ZIS_6_, c) FTO/ZIS_12_ and d) TiO_2_NT/ZIS_12_ photoanodes before and after annealing at 300, 400 and 500 ^o^C recorded without illumination in 0.5 M Na_2_SO_4_.





**Fig. S7.** LSV curves of FTO/ZIS_6_ photoanode in different electrolytes.

**Table S3.** Photoelectrochemical performance of photoanodes.

| Material | The highest photocurrent [mA cm^-2^] | | | Photocurrent drop after 600 s of exposure [%] | | |
| --- | --- | --- | --- | --- | --- | --- |
|  |  |  |  | pH=1 | pH=7 | pH=14 |
|  | ph=1 | pH=7 | pH=14 |  |  |  |
| **FTO/ZIS_6_** | - | 0.24  (at 1.62 V vs. RHE) | - | - | - | - |
| **FTO/ZIS_6_-O_300_** | - | 0.19  (at 1.53 V vs. RHE) | - | - | - | - |
| **FTO/ZIS_6_-O_400_** | - | 0.08  (at 1.62 V vs. RHE) | - | - | - | - |
| **FTO/ZIS_6_-O_500_** | - | 0.07  (at 1.62 V vs. RHE) | - | - | - | - |
| **FTO/ZIS_12_** | - | 0.29  (at 1.62 V vs. RHE) | - | - | - | - |
| **FTO/ZIS_12_-O_300_** | - | 0.23  (at 1.53 V vs. RHE) | - | - | - | - |
| **FTO/ZIS_12_-O_400_** |  | 0.60  (at 1.53 V vs. RHE) | - | - | - | - |
| **FTO/ZIS_12_-O_500_** | 0.84  (at 0.31 vs. RHE) | 1.21  (at 1.62 V vs. RHE) | 0.87  (at 1.64 V vs. RHE) | 52 | 75 | 50 |
| **TiO_2_NT/ZIS_6_** | - | 0.04  (at 1.62 V vs. RHE) | - | - | - | - |
| **TiO_2_NT/ZIS_6_-O_300_** | - | 0.26  (at 1.62 V vs. RHE) | - | - | - | - |
| **TiO_2_NT/ZIS_6_-O_400_** | - | 0.16  (at 1.62 V vs. RHE) | - | - | - | - |
| **TiO_2_NT/ZIS_6_-O_500_** | - | 0.05  (at 1.62 V vs. RHE) | - | - | - | - |
| **TiO_2_NT/ZIS_12_** | - | 0.03  (at 1.32 V vs. RHE) | - | - | - |  |
| **TiO_2_NT/ZIS_12_-O_300_** | 3.02  (at 0.31 V vs. RHE) | 0.50  (at 1.62 V vs. RHE) | 1.23  (at 0.34 V vs. RHE) | 37 | 21 | 41 |
| **TiO_2_NT/ZIS_12_-O_400_** | - | 0.36  (at 1.62 V vs. RHE) | - | - | - | - |
| **TiO_2_NT/ZIS_12_-O_500_** | - | 0.02  (at 1.62 V vs. RHE) | - | - | - | - |

**
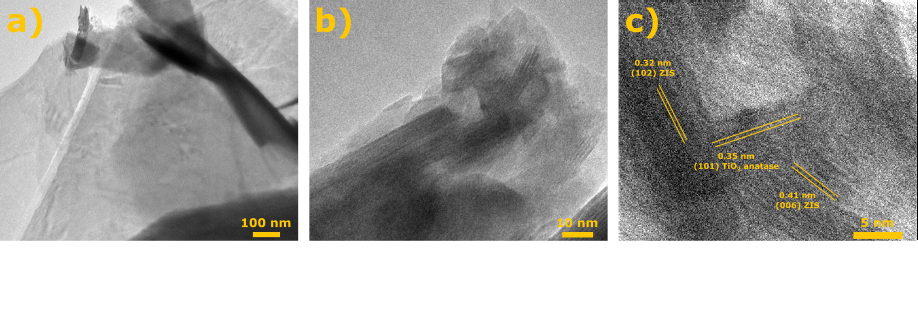
Fig. S8**. a) and b) TEM images of TiO_2_NT/ZIS_12_-O_300_, c) HRTEM image of TiO_2_NT/ZIS_12_-O_300_


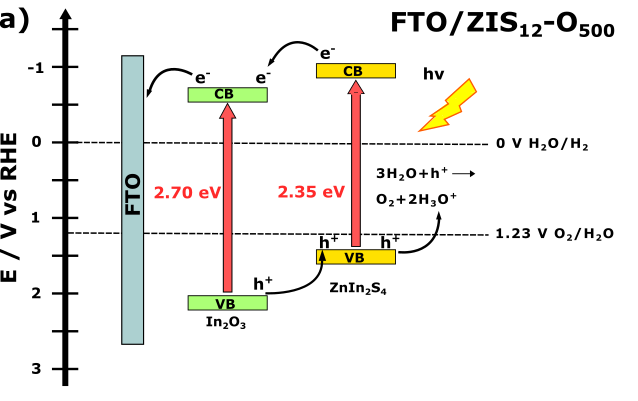

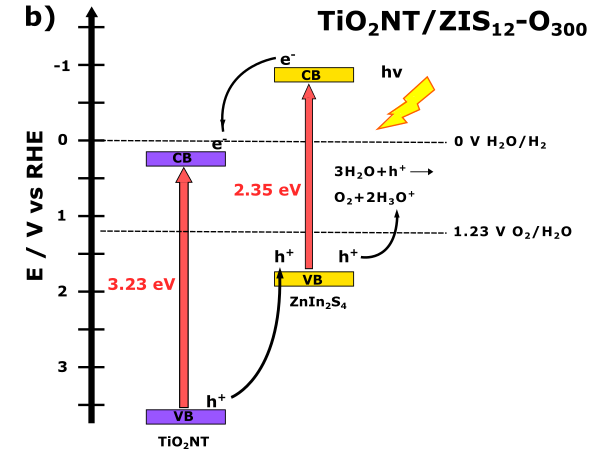


**Fig.S9.** Schematic mechanism of the electron and hole transfer in a) FTO/ZIS_12_-O_500_ and b) TiO_2_NT/ZIS_12_-O_300_ photoanodes.





**Fig. S10.** Mott-Schottky plots and CV curves (0.5 M Na_2_SO_4_) of a) TiO_2_NT and b) FTO/ZIS_12_; Tauc plots of c) TiO_2_NT and d) FTO/ZIS_12_ photoanodes


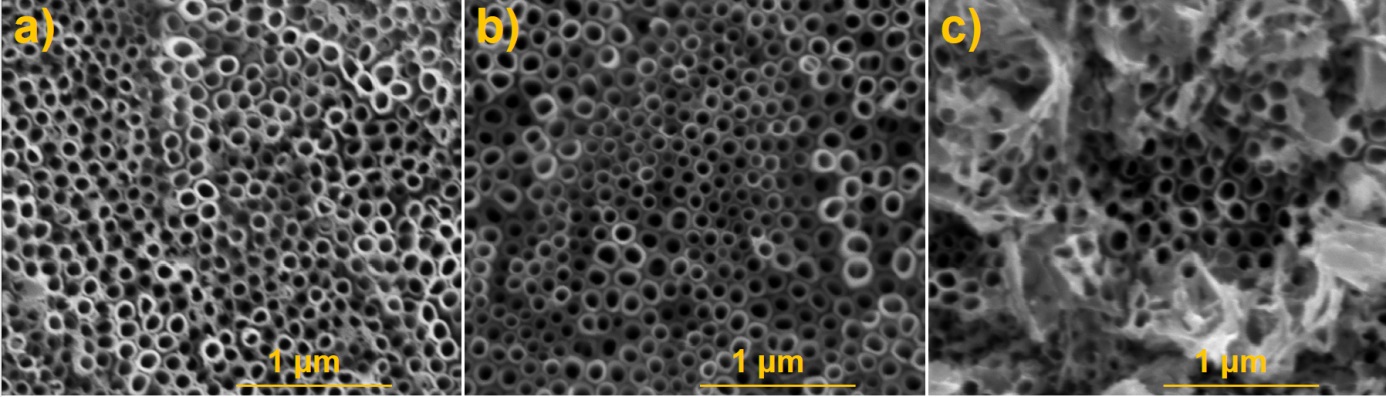


**Fig. S11.** SEM images of a) pure TiO_2_NT and b) TiO_2_NT/ZIS_12_ after removing the active layer and c) TiO_2_NT/ZIS_12_-O_300_ after removing the active layer.





**Fig. S12.** XRD patterns of FTO/ZIS_12_-O_500_ and TiO_2_NT/ZIS_12_-O_300_ before and after 2h of illumination.


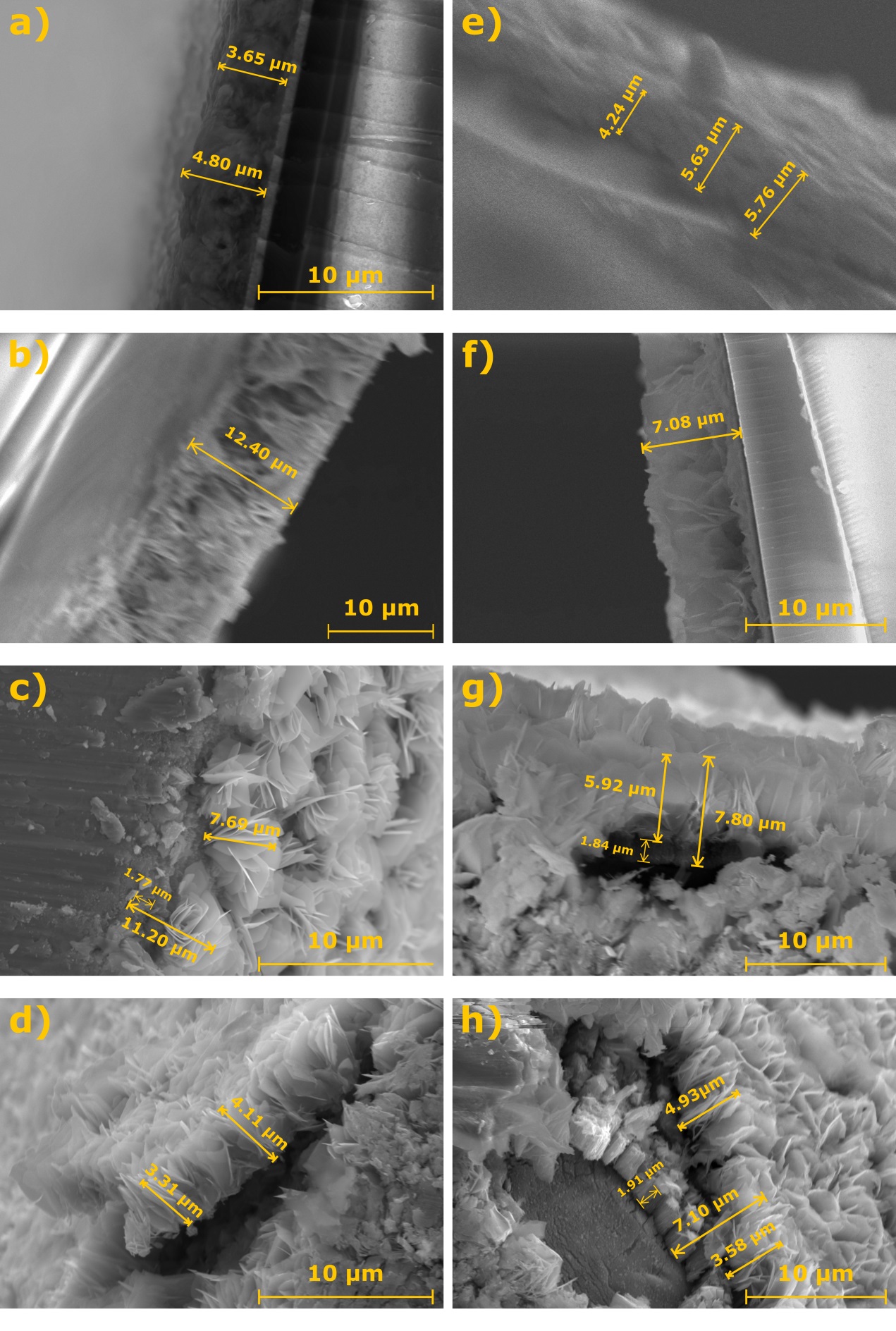


**Fig. S13.** Cross-section SEM images of a) FTO/ZIS_12_, b) FTO/ZIS_12_-O_500_, c) TiO_2_NT/ZIS_12_, d) TiO_2_NT/ZIS_12_-O_300_ before and e) FTO/ZIS_12_, f) FTO/ZIS_12_-O_500_, g) TiO_2_NT/ZIS_12_, h) TiO_2_NT/ZIS_12_-O_300_ after 2h illumination.

**References**

1. Siuzdak, K., Szkoda, M., Sawczak, M. & Lisowska-Oleksiak, A. Novel nitrogen precursors for electrochemically driven doping of titania nanotubes exhibiting enhanced photoactivity. *New J. Chem.* **39**, 2741–2751, 10.1039/c5nj00127g (2015).
2. Uvarov, V. & Popov, I. Metrological characterization of X-ray diffraction methods for determination of crystallite size in nano-scale materials. *Mater. Charact.* **58**, 883–891, 10.1016/j.matchar.2006.09.002 (2007).
3. Kubelka, P. & Munk, F. Ein Beitrag zur Optik der Farbanstriche. *Zeitschrift für Technische Physik* **12**, 593-601, (1931).
4. Liu, C. *et al.* Design of Core–Shell-Structured ZnO/ZnS Hybridized with Graphite-Like C_3_N_4_ for Highly Efficient Photoelectrochemical Water Splitting. *Adv. Mater. Interfaces* **4**, 1–11, 10.1002/admi.201700681 (2017).
